# Nephron Sparing Treatment (NEST) for small renal masses: understanding patients’ and clinicians’ experiences of a cohort-embedded randomised control trial

**DOI:** 10.1186/s40814-026-01833-7

**Published:** 2026-05-13

**Authors:** Veronica Ranieri, Hannah Warren, Joana B. Neves, Joseph Santiapillai, Nicola Rode, David Cullen, Graham Munneke, Miles Walkden, Prasad Patki, Ravi Barod, Faiz Mumtaz, Michael Aitchison, Elena Pizzo, Norman R. Williams, William H. Wildgoose, Kurinchi Gurusamy, Mark Emberton, Axel Bex, Maxine G. B. Tran

**Affiliations:** 1 Research Department of Clinical, Educational and Health Psychology, University College London, London, UK; 2 Division of Surgery and Interventional Sciences, University College London, London, UK; 3 Specialist Centre for Kidney Cancer, Royal Free Hospital, London, UK; 4 Department of Interventional Radiology, University College London Hospital, London, UK; 5 Department of Applied Health Research, University College London, London, UK; 6 Patient Representative, London, UK

**Keywords:** Feasibility studies, Nephrectomy, Cryotherapy, Kidney neoplasms

## Abstract

**Background:**

To understand the perspectives and experiences of patients and healthcare professionals regarding the trial design, recruitment and randomisation process to a novel cohort-embedded RCT design for Nephron Sparing Treatment (NEST) for small renal masses.

**Methods:**

Participants were adult patients with a small renal mass (< 4 cm) randomised to receive either percutaneous cryotherapy (CRO) or robot-assisted partial nephrectomy (PRN), and clinicians involved in a trial comparing the two treatments. Semi-structured interviews followed a phenomenological approach and were transcribed verbatim and analysed using thematic analysis.

**Results:**

Interviews were conducted with 37 patients and 10 clinicians. Wanting to ‘give back’ influenced participants’ decisions to take part in the trial. Most patients expressed that they had a positive and informed experience of the trial. Patients who accepted randomisation to cryotherapy (experimental intervention) were influenced by information provided regarding a potentially shorter recovery timeframe, length of hospitalisation, and lower risk of complication. Those who were randomised to receive partial nephrectomy (standard of care intervention) stated that they felt less anxious receiving ‘non-experimental’ treatment and felt safer in the knowledge that the tumour was removed. Clinicians expressed that the trial presented an opportunity to work collegially in identifying the best treatment for patients presenting with SRMs, though highlighted that limited consultation time was a challenge to recruitment.

**Conclusions:**

Findings support the potential feasibility of a future multicentre trial comparing percutaneous cryoablation to robot-assisted partial nephrectomy in the treatment of small renal masses. The study’s design, process and intervention were accepted by most, though not all, participants.

**Trial registration:**

ISRCTN18156881

## Key messages regarding feasibility

1. What uncertainties existed regarding the feasibility? The feasibility of a trial comparing the two interventions for a small renal mass and participants’ and clinicians’ understanding and perspective a novel trial method.
2. What are the key feasibility findings? Participants reported a positive experience of the trial though memory recall was a factor. Limited consultation time was a challenge for clinicians recruiting to the trial.
3. What are the implications of the feasibility findings for the design of the main study? A larger multi-centre trial comparing percutaneous cryoablation to robot-assisted partial nephrectomy in the treatment of small renal masses appears feasible.

## Introduction

The incidence of small renal masses (SRMs) (< 4 cm in maximal diameter) continues to rise [1, 2], in part due to incidental diagnosis as a result of increased use of medical imaging [3]. Treatment options include partial or radical nephrectomy, thermal ablation and active surveillance, based on the characteristics of the disease and patient [4].

Whilst nephron-sparing partial nephrectomy is the current standard of care for SRMs, less invasive ablative options, such as cryoablation, are recommended in frail or comorbid populations [5]. Percutaneous cryoablation (CRO) is associated with fewer complications and faster recovery compared to robot-assisted partial nephrectomy (RPN); however, it does not allow for a definitive histological analysis [5]. However, there is no high-level evidence comparing the two forms of treatment. Previous standard randomised controlled trials in this space did not complete recruitment; for example, SURAB (Study comparing ABlation with active SURveillance, in the management of incidentally diagnosed small renal tumours: a feasibility study, ISRCTN31161700) recruited 5 patients out of a pre-defined milestone target of 32 [6]. The reasons for non-completion were reported to be due to several factors, including delays in obtaining regulatory approvals, unwillingness for treatment decisions to be decided by chance, delays in treatment in the ablative arms due to lack of radiological capacity and insufficient trial duration.

Alternative pragmatic trial designs such as the cohort embedded RCT (ceRCT) or Trials within Cohorts (TwiCs) have been used to address recruitment challenges and have been used successfully in other disease settings such as in chronic obstructive airways disease and colorectal cancer [7, 8]. The Nephron Sparing Treatment (NEST) for small renal mass feasibility study (ISRCTN18156881) was performed to assess if using a ceRCT design could facilitate recruitment to a clinical trial comparing CRO to RPN and successfully met its primary outcome of recruiting 50 patients to the embedded RCT [9], with 46/50 (92%) accepting their allocated treatment (84% in the CRO arm and 100% in the RPN arm). ceRCTs incorporate a two-stage informed consent procedure that consisted of a combined consent for cohort participation and randomisation, with a separate intervention consent for those randomised to the CRO arm [10]. The first stage requires participants to consent for cohort participation, i.e. for consent for long-term follow-up, completion of patient reported outcome measures and questionnaires, bio-banking of samples (optional), participation in future trials as a standard care control group without the need for further consent, and future randomised invitation to consider new interventions or tests [11]. In the second stage, cohort participants fulfilling eligibility criteria for the embedded RCT and who are randomised to the intervention arm are approached to consider the intervention (second stage consent). A qualitative workstream was embedded as part of the NEST feasibility trial, and in-depth interviews were performed with patients and clinicians who were involved in the trial. The aim of this study is to explore the acceptability of the trial design, and the experiences and perspectives on recruitment and understanding of randomisation and consent, with a view to informing the design and conduct of a future multicentre trial.

## Methods

### Participants

Participants consisted of (i) adult patients managed at a single tertiary-referral hospital for a small renal mass (< 4 cm) and randomised to receive CRO or RPN in the NEST trial and (ii) clinicians involved in the recruitment or treatment arms of NEST [11]. Participants were excluded if they were non-English speakers, were unable to consent to be interviewed or had advanced disease (N1 and/or M1 on TNM staging), as per the NEST trial [11]. Participants were interviewed between November 2019 and December 2021. The interview period was extended due to COVID-19-related disruption. Interviews with patients were conducted post-treatment for five main reasons: (1) to allow participants to reflect on their experience after their journey on the entire care pathway; (2) to avoid adding research burden at a vulnerable time for patients; (3) to prevent interviews from potentially impacting patients’ preferences to treatment; (4) to capture whether the design was feasible in retrospect and (5) for practical feasibility issues (e.g. prevent interview cancellations should there be treatment changes). Participants were broadly consented at entry to the study Clinicians were purposively sampled according to specialty and type of involvement within the trial (e.g. those who were involved in the day-to-day running of the trial and those who provided clinical input only) to ensure that a breadth of viewpoints was included.

### Setting

Interviews took place via telephone or virtual video platform (i.e. Zoom, Teams), as per participants’ preferences, particularly in the context of the COVID-19 pandemic. Interviews took place after patients had received their treatment.

### Recruitment

Consecutive eligible patients were identified at weekly multidisciplinary team (MDT) meetings before being approached for recruitment to the NEST trial [11]. Those who agreed to take part in the interviews were sent a copy of the information sheet and asked to give verbal informed consent, recorded on a digital voice recorder.

### Analysis

This study employed a phenomenological study design as a way of understanding the experiences of participants and the meaning that participants attributed to these experiences. This was done by conducting in-depth semi-structured interviews with open-ended and neutral questions designed to elicit reflective and descriptive narratives of patients’ lived experiences and perceptions, rather than solely factual-based recollections. Interviews were conducted by a research psychologist with clinical experience, and reflexivity was actively considered. The interviewer used open-ended prompts and utilised both a reflective journal and peer supervision to bracket and check own assumptions and subjectivities. Interviews were transcribed verbatim by Essential Secretary Ltd and coded using NVivo Release 1.6.2 as a Computer Assisted Qualitative Data management system [12]. Transcripts were analysed using Braun and Clarke’s (2006) thematic analysis, with features of narrative analysis [13, 14]. This approach was used to generate, review and define themes within the interview transcripts with member checking. Discussion regarding the themes took place within the research team.

### Ethics approval

This study was approved by the Health Research Authority NHS and Health and Care Research Wales (HCRW), Research Ethics Committee reference 19/EM.0004. All participants provided informed consent to participate in the study.

## Results

A total of 50 patients were recruited to the NEST ceRCT of whom 37 (74%) consented to be interviewed about their experiences (18 CRO arm, 19 RPN arm) for feasibility. The majority of those who did not participate in the interviews were uncontactable (e.g. change of contact details, not answering recruitment contact). One individual had a significant language barrier that prevented him from taking part whilst another expressed that they did not want further confidential information to be held about her within that national health service. Interview participants had a median age of 60.5 years (range 47–77 years), and 43% were women. Interviews took place, on average, 1.3 years after the patient received their procedure. This was due to communication delays between the clinical and research teams during the COVID-19 pandemic, difficulties experienced by the interviewing researcher who was also employed as a mental health clinician during the pandemic and difficulties in establishing contact with multiple participants.

Ten clinicians were interviewed about their experiences of the trial (6 urologists, 2 interventional radiologists, 2 clinical nurse specialists). Three of these clinicians directly recruited participants to the trial and the remainder were involved in the management of participants.

The following themes emerged from patient participants: (1) reasons for participation; (2) barriers to participation; (3) information-sharing and informed consent; (4) perceptions of randomisation and choice; and (5) preferences for treatment (Table 1). Themes raised by clinicians included perceptions related specifically to the treatment arms, experiences of the trial, and suggestions for future directions for clinical research and practice. For quotations for each theme, please see Table 2.

**Table 1:** Patient and clinician themes regarding the trial

Themes	Patients	Clinicians
*Reasons for taking part/purpose of the trial*		
	Altruism	Advancing science
	To give back	Trial new design
	Advancing science	Understand the patient’s perspective
	Curiosity	
	Impact on treatment received	
*Deterrents to taking part*	Concerns regarding privacy	
	Inflexibility	
	‘Trial’	
*Information & consent*		
	Differing understanding of trial	
	Temporality and pace	
	Preferences for detail	
	Accessibility of information sheet	
	Format of information	
	Memory recall	
	COVID-19-related delays	
*Perceptions of randomisation and choice*		
	Recognition and agreement	
	Disagreement or refusal	
	Unawareness	
	Trust	
	Personal agency	
*Preferences for treatment*		
	Risk	
	Invasiveness	
	Prior experiences	
	‘Untested’ treatment	
*Experiences of the trial*		
	Involvement	Disparities in consent rate
	Support	Time pressures
	Organisation	Administrative burden
	COVID-19 related delays	Pressures within MDT
		Support between disciplines
*Future directions for clinical research and practice*		Availability of information prior to MDT

**Table 2:** Quotations from patient and clinicians’ accounts

Patients	Quotations
*Reasons for taking part in the trial*	‘I was so grateful to receive the treatment that I was receiving and the level of service from the NHS that I was receiving and everyone, just everyone’s kindness towards me, when you’re going through something quite traumatic that I was just happy to give anything back and doing my little thing to help someone else or the NHS.’ Participant 0043, Male, 50 s, randomised to cryotherapy ‘I think if these things are being done for the right reasons, and in 20 years it saves somebody else's life, or there's more information to allow them to maybe save more lives, then that's a good thing, surely.’ Participant 0188, Male, 70 s, randomised to partial nephrectomy ‘I was more than happy to participate. It was no great hardship or anything from my point of view and obviously it would be beneficial for other people in the future and for the research, so I was happy to help out.’ Participant 0057, Male, 70 s, randomised to partial nephrectomy ‘It was all, sort of, concurrent with any treatment I was receiving, so it was never an issue or a problem. I might have had to wait around for an extra five/ten minutes to do a blood test or anything like that, but that was it, it was never an inconvenience’ Participant 0043, Male, 50 s, randomised to cryotherapy ‘I’ve participated in them [trials] before, I don’t know if it’s just me but they tend to look after you a bit better.’ Participant 0041, Male, 50 s, randomised to partial nephrectomy ‘My mum died of cancer and I remembered a couple of people who died of cancer… if I could do anything in the research to be able to make it easier or make it more survivable for the future people then that’s fine, that’s why I was interested in helping out’. Participant 0055, Male, 60 s, randomised to cryotherapy ‘It was again just speaking to a really good surgeon who explained everything that made me decide to go through with that. That’s quite important in the procedure to be told he’s confident about what they are asking you to do.’ Participant 0008, Male, 60 s, randomised to cryotherapy
*Deterrents to taking part*	‘The Royal Free are very keen on saying to me, come up there and have a face-to-face meeting. But it’s like a two-hour journey for me, to there, and back, which is … and it’s a very hard journey, sort of going into London. And I think that they should be able to give you options of having more online meetings, just in general.’ Participant 0175, Female, 50 s, randomised to cryotherapy ‘The word trial, it’s like this is … the first time it’s been done. What if something goes wrong? That was the scary bit, but… I realised that when things in the NHS or medical field say trial, they actually mean they’ve done it a few times before.’ Participant 0089, Female, 60 s, randomised to cryotherapy
*Information & consent*	‘At the first meeting I’m not sure I initially understood what the trial was for. It might have been explained, but it was explained very quickly and I thought it was just a procedural thing, to do with people’s experiences. I didn’t understand it was a surgical trial to begin with at the very beginning… there was no way that I got anywhere near surgery in the follow up sessions without fully understanding… what the procedure was and what I was doing and why I was doing it.’ Participant 0001, Female, 60 s, randomised to cryotherapy ‘I would say everything was done really well. They was friendly, the way they spoke to you, it wasn’t, sort of, patronising, it was, you know, it was very good, very good.’ Participant 0037, Male, 50 s, randomised to partial nephrectomy ‘I had a conversation with someone who was sat next to me, explaining to me what the situation was with it. So, I didn’t need to read through a ten-page pamphlet to understand what somebody's saying to me while we're having a conversation.’ Participant 0188, Female, 50 s, randomised to partial nephrectomy ‘Part of me doesn’t want to know anything, I just want to get it over and done with.’ Participant 0167, Male, 70 s, randomised to cryotherapy ‘That NEST information was quite a lot of writing. I’m a good reader, you know, I’m used to reading a lot of text, but I think for a lot of people they would find it better, and easier to absorb, if it was done in an easy read format.’ Participant 0175, Female, 50 s, randomised to cryotherapy ‘For me, personally, I will get paperwork and I don’t always read it to be honest. Yet if there was some sort of video presentation, you can sit and watch… I could watch for ten, fifteen minutes… I would quite happily watch it. But to plough through the paperwork, just on the trial, I’d had the paperwork about the cryoablation treatment, itself, about my cancer treatment, and other things as well, to get in with.’ Participant 0119, Male, 50 s, randomised to cryotherapy ‘The first conversation I ever had about it was over the phone when he told me that I definitely had cancer… at the time as I said COVID was very prevalent and I wasn’t happy to go into a hospital and meet anybody face to face… so that’s when he told me about the trial and initially, I agreed to it, but I wasn’t thinking about it, I was just trying to take in the fact I’ve just been told I had cancer. I just wasn’t taking in what [the nurse] was saying. I think giving somebody a cancer diagnosis and then giving them information about a trial, it’s like being told you have cancer is such a shock no matter how small it is… the word is so frightening and to be told that then I don’t think your brain processes everything.’ Participant 0137, Female, 50 s, randomised to cryotherapy ‘I can’t really remember, because I’ve been in so many times with my throat issue, my shoulder issue, I’ve been in so many different things… I couldn’t really tell you’ Participant 0119, Male, 50 s, randomised to cryotherapy ‘They told me about the study and told me it would involve questionnaires, told me it would involve extra blood tests, told me it would involve an extra tissue sample at the point of biopsy which I said, I said they could take, I was happy for them to use any of my tissues as part of the study.’ Participant 0015, Female, 60 s, randomised to partial nephrectomy ‘The way they explained it [cryotherapy] was that it has been going for 10 years, 400 people have had this surgery, and in a way, getting feedback from people like myself, who was going through this surgery, would be able to help the health profession know, and understand how effective the surgery is, if there are any side effects.’ Participant 0121, Female, 60 s, randomised to cryotherapy
*Perceptions of randomisation and choice*	‘I saw the doctor and she told me the result and then she told me the options and she told me that I’d been randomised. I didn’t even understand what she meant by that, that I’d been randomised to have the operation. I should have really asked her, but I just so happy that they were going to go ahead and do the operation, but she didn’t really explain what she meant by, you know, that I’d been randomised. … Well, if I think about it, it sounds more like a lottery thing, yes [chuckling]. You put names in a hat and you pick out one for this and one for that. I was just so worried in case they changed their mind, so, I didn’t really delve into it.’ Participant 0125, Female, 60 s, randomised to partial nephrectomy ‘Now I was told I was picked at random… I can’t remember which visit it was, when they said oh by the way, you’ve been picked at random, and are you happy to go along, and I went yeah, I’m fine, I’m quite happy to go along with this… I had a bit of a conflict. I thought oh, why are they just picking people off from a computer, not asking them? So that was my first reaction. It shocked me a bit to think why should a computer pick me up randomly… but they were gracious, they were saying you’ve been picked randomly, how do you feel about it? It wasn’t like you’ve been picked randomly, and you are going to do it. So I was still given the choice, you know, you’ve been picked randomly, but you can always say no.’ Participant 0121, Female, 60 s, randomised to cryotherapy ‘Yeah, they basically said that which of the treatments I got was randomly selected, but they also said if I wasn’t happy with my randomly selection, of course, I could change my mind and go for the other treatment.’ Participant 0113, Male, 50 s, randomised to partial nephrectomy ‘I mean maybe for some people if you took part in the study they randomly allocated you, but that was never going to suit me because I didn’t want to be randomly allocated, I wanted to have the surgery. If I’d accepted randomisation I would have gone for whatever I’d been randomised to but I wouldn’t have accepted randomisation. That would have made me not want to be in a trial if they said I had to.’ Participant 0015, Female, 60 s, randomised to partial nephrectomy ‘I don’t know. Is it entirely random though? Don’t they put in patient factors, don’t they, before they randomise it? I’m not sure entirely how that works.’ Participant 0138, Female, 60 s, randomised to cryotherapy
*Preferences for treatment*	‘I'd already had that surgery before, four years ago. So, they sort of said, you can either have done what you'd had done before, or we have this other thing that could be potentially used. So, then I went home and looked it all up, and decided that I’d rather have the surgery. Yeah, because I knew what to expect… I got over it well. Although it was painful, obviously, it’s a major operation, I thought I healed pretty well, and everything's been alright since then.’ Participant 0175, Female, 50 s, randomised to cryotherapy ‘Yes, do that, because that doesn’t mean surgery, as in scars, stitches and horrific aftermaths for me. I don’t do well with anaesthetic, or I don’t heal well either, with scars. So that sold it to me, even though she was fantastic and went through absolutely every other option there was, I chose the cryo.’ Participant 0089, Female, 60 s, randomised to cryotherapy ‘They didn’t sort of like try and frighten me off of that option. They didn’t say oh like “We can do the surgery, sort of like the freeze, which is 100% safe and we can do the surgical which is 50% safe”. It was nothing like that. It was very much sort of like telling me the facts about both options. And then just explaining the impacts, that it may take longer for my renal function to recover from the result of the surgical procedure than it would from the freeze procedure, the cryoablation.’ Participant 0130, Male, 50 s, randomised to cryotherapy ‘I said to her… what would you do, if this was you or your family member what would you recommend and… she said the surgery route is kind of a tried and tested method I suppose and very much it’s out then, they’ve removed it. Whereas the cryo I suppose is fairly new technology and we were concerned with the damage the cryo could cause to the rest of the kidney, although the incisions are damaging… I asked her, I said which is the best and she said I’m probably the wrong person to ask because I’m a surgeon, and I’m going to have a biased view but it’s, she could influence our decision.’ Participant 0041, Male, 50 s, randomised to partial nephrectomy
*Experiences of the trial*	‘I had 100% faith in the doctors and what they were doing and also the nurse as well who, I’ve got to say, was absolutely amazing in reassuring me and helping and being available. I know she had to deal with so many people at the same time, but, to make you feel like you’re the only person they’re treating, that’s how I felt with the nurse as well, it was just … everything just went smoothly.’ Participant 0043, Male, 50 s, randomised to cryotherapy ‘I think from the moment I said yes I’ll do the trial; things went very fast. I mean within eight weeks of saying yes, I’d had the surgery… this is very quick. I didn’t feel railroaded, but I did comment to my wife a couple of times things are moving very quickly. Not that I couldn’t cope with the pace of it, but things did move very quickly from the moment I said yes.’ Participant 0001, Female, 60 s, randomised to cryotherapy ‘I accept that this is the National Health Service, and time is at a premium, and I’m happy to have to bend their schedule, because you know, this is a quality service I’m basically getting for free. But having said that, you know, it is slightly … it makes one slightly apprehensive to have to chase and have to … you know, people not having got back to you.’ Participant 0113, Male, 50 s, randomised to partial nephrectomy ‘I was introduced to… the nurse who would support me, and I was told, oh, you know, she’s the kidney cancer nurse. She’s there to support you, and she was very nice, and friendly there, and she gave me her business card and that. It had two phone numbers on it, one of them had a voicemail which I left various voicemails on and never got a reply. The other phone number didn’t exist. And, then I emailed her twice, and never got a reply. So, that was really unhelpful, and quite distressing to be honest.’ Participant 0175, Female, 50 s, randomised to cryotherapy
**Clinicians**	**Quotations**
*Purpose of the trial*	‘We know that surgery is an invasive procedure, and even the best surgeons in the world will still have an element of complication risk and will have complications that need further treatment, investigation or in some very sad cases death from surgery. So I just don’t think that we should be just offering surgery if there are equivalent alternatives available, and it should be an informed choice. But we don’t have the evidence. We need the evidence, we need the evidence that it’s acceptable. We need the evidence that it works from the patient and from the clinician perspective. That’s the whole purpose of this research, whole purpose of NEST really.’ CL10, Surgeon ‘If we could get some feedback on what the patients … because I mean I think that's vital isn’t it really, to delivering something? Because is there something that they feel that we hadn’t told them beforehand that if we had done would have massively changed what they chose to do? Did they feel they had a choice?’ CL07, Radiologist
*Perceptions regarding treatment*	‘Well the data doesn’t support that at the moment because the longest follow up that we have in the future, if I’m not mistaken is seven years. The patient is going to have a life expectancy of 45 years, that’s number one. Number two, as I said to you surgical treatment provides you with a histology that your margin is negative and the tumour is out. I can tailor my follow up with just simple non-radiation-based imaging after I’m relatively certainly that the tumour is controlled and gone whereas when you put in needles and you freeze it, it takes a long time for the body to dissolve that, form a scar and the only way that you can say that the recurrence is there or not, is to do a contrast CT scan. These are factors you have to account for. You can’t do CT scans on a patient for over 20 or 30 years, how do you account for that? The patient has to have real faith in the treatment. We don’t take biopsies after cryotherapy of a relatively normal kidney… because you won’t be able to tell the difference, whether it’s necrosed tissue, whether it’s a response from the body, a vascularity because the body is repairing the damaged tissue which is very common.’ CL02, Surgeon
*Experiences of the trial*	‘My first aim is to reassure them. I think people who tend to be reassured, they probably are more likely to consider being part of this as well because there’s less on their minds. Most of the time they have received this incidental diagnosis, one or two weeks before coming to clinic and then in the clinic appointment they have a bit of a more comprehensive view about the health issues and hopefully some reassurance and then, at the end of the clinic appointment I mentioned research and I see if they are willing to take part in it. If they say immediately no, then I don’t push on it. If they let me speak then I’ll give them a bit more detail and then discuss with them if they would be willing to participate.’ CL03, Surgeon ‘If they don’t have any preparation before they come then their experience is really bad because they, no one has ever made the word cancer before and they’re coming to a cancer clinic. So, although I think it relates to very much general patient experience in general but if you’re looking at research, if someone is coming, they don’t know why they are coming, adding a research element into a shocking conversation then it will affect recruiting. So, I think we just have to have a standard in our practice that patients are prepared in advance.’ CL04, Nurse ‘I think both… may feel a bit threatened I guess. So, surgeons might feel as if this study might take away their skill, their purpose. And cryo, interventional radiologists might feel the same way. But it’s just, I think it’s unfounded. These concerns are unfounded. I think everyone’s a professional and you need to have faith in what you’re doing, and to do that you have to have evidence. And I’m pretty sure that this is not going to impact on anyone’s career or anyone’s livelihoods or anything. It’s just going to tell us exactly which patients are going to benefit from which treatment, and what we can offer these patients. And it’s trying to reassure my colleagues that I’m not trying to destroy, I’m not trying to take away their bread and butter or their purpose in the NHS. It’s going to help all of us. It’s going to help us guide our consultation.’ CL10, Surgeon

### Patients

#### Reasons for participation in NEST

The most frequent motivator for participation was altruism and wanting to ‘give back’ to the health service and clinicians. Some participants expressed an active interest in research, with some reporting prior positive experiences or encouragement from family or friends with such experiences. Some participants described initial anxiety about being a ‘guinea pig’ but were reassured after counselling by clinicians experienced with the study intervention.

The pragmatic trial design facilitated participation, with patients conveying that the ceRCT did not require them to travel or attend extra appointments, and that they did not find the research team’s surveys and request for extra urine or blood samples onerous. Some communicated a belief, imparted by relatives, that their participation in the trial would improve the treatment they received via a more experienced team that provided greater attention to their needs. Receiving clear information that outlined the potential risks of participation and not feeling pressured to take part fostered trust and facilitated recruitment.

### Barriers to participation in NEST

A minority of participants highlighted that the term ‘trial’ initially deterred them from taking part as it implied a novel and yet untested intervention. One expressed that they had initial concerns that the team may prioritise their research needs over the patient’s clinical needs. Regarding interviews, two individuals expressed concerns regarding anonymity and privacy knowing their information would be shared with the research team. Some expressed a preference for telephone rather than video/in-person interviews for privacy reasons.

### Information-sharing and informed consent

Most participants spoke of receiving verbal information about the trial during a clinic visit or telephone call with a clinician, followed by written information prior to giving consent. Most stated that the information provided was clear, detailed and delivered in a friendly manner. Preferences regarding the amount and depth of information about the trial and related interventions differed between individuals. Patient information sheets were generally considered too long, and an easier-to-read format or video would have been preferred, such as a video. Those randomised to RPN had access to an information video given as part of routine care which was referred to positively. Some participants spoke of searching the internet later within their own time to complement and independently verify the information received.

### Perceptions of randomisation and choice

The majority of participants accepted their randomised treatment and spoke of trust in their clinical team. One participant reported feeling comfortable to ask to change their allocated treatment, while another reported they would not have felt able to refuse randomisation for fear of experiencing further delays to treatment. Approximately half of those randomised to receive cryotherapy stated that they were aware of the randomisation of treatment (*n* = 9). Four individuals stated that they had not understood that they were randomised, three did not recall and two stated that they were offered a choice of treatment.

### Preferences for treatment

At interview, most participants expressed a preference for one treatment or another. Almost all of those who were randomised to receive CRO expressed that they had a preference for CRO, describing it as a quicker, less intrusive, scar-free procedure that required a shorter hospitalisation and recovery time. Some participants stated a preference for new technology and novel treatments. The only negative perception of CRO was that of residual cancerous cells. An individual who declined randomisation to CRO stated a preference for surgery as they had prior positive experience of surgery.

Participants randomised to receive RPN expressed a preference for surgery citing concerns regarding cryotherapy’s oncological effectiveness, risk of damage to surrounding kidney tissue and describing it as an ‘unproven’ treatment.

### Clinicians

#### Trial experience

The majority of interviewed clinicians spoke positively of contributing to a novel methodological trial design with the potential to improve recruitment and the opportunity for surgical and interventional radiology teams to work collegially, rather than competitively, to identify the best treatment for patients presenting with SRMs.

Three of the clinicians interviewed directly approached, recruited and consented patients to participate in the ceRCT. They reported a positive experience overall, with most patients consenting to be part of the trial. However, other non-recruiting clinicians spoke of limited consultation time being a challenge to the consent process, and prioritised clinical care of patients over research activity. There were conflicting views on sending participant information sheets to patients prior to their consultation, as some patients may not be aware of their diagnosis.

Screening patients at a weekly multi-disciplinary team/tumour board meeting played a central role in establishing and maintaining a positive research culture during the trial. Discussions regarding when CRO and RPN were technically feasible led to interventional radiologists reporting a shift from being perceived as ‘technicians’ to colleagues with complementary skill sets. A minority of surgeons felt that the scope of their practice may be under threat.

## Discussion

The main findings from this study were that recruitment to a ceRCT was feasible and acceptable to patients and clinicians. Patients reported participation on the basis of altruism and conditional altruism, meaning willingness to participate for the benefit of others but in the knowledge that trial participation brings personal benefit or absence of harm [15]. Participants also reported the pragmatic study design minimised the burden of participation as the trial was embedded into routine clinical care, with no additional visits, and participants not selected for randomisation continued with standard care.

Study participants described having ‘chosen’ to accept the treatment option that was offered to them (CRO or PRN) despite it being allocated at random as part of the ceRCT. In general, patients who accepted to receive CRO described the positive attributes of CRO and downplayed those of RPN, and vice versa. This may be explained by choice-supportive bias in which people tend to retrospectively ascribe positive attributes to what they perceive as their previous choice or low decision regret [16]. It may also suggest that some participants did not fully understand or recall the process of randomisation, thus potentially limiting the validity of the informed consent process. Nonetheless this finding suggests that study participants were content with the treatment offered as part of the trial. It has been observed previously that participants who agree to be randomised in trials may express preferences for particular treatments [15, 17]; however, as interviews in NEST were conducted after completing the study intervention, it is not known if participants had different preferences prior to random allocation.

Clinicians reported a desire to contribute to a novel trial design, but some found the time allocated for each patient consultation insufficient to approach potential participants. Allocation of additional clinic time for eligible patients may facilitate recruitment in future studies. Future studies may benefit from providing information that is interactive or videographic in nature to help facilitate understanding in patients [18], and the inclusion of references in information sheets. As the word ‘trial’ caused uncertainty regarding participation, it may be beneficial for future research to use an alternative word and for clinicians to ensure that patients understand what randomisation entails.

The two-stage consent process incorporated into ceRCTs/TwiCs has led to discussions centring on the ethics of the first stage ‘broad consent’ that includes randomisation to a standard care control arm without further explicit consent in the second stage [19, 20]. A recent cross-sectional survey on participants in TwiCs in breast and rectal cancer asking these specific questions showed that recollection of having given broad consent to randomisation was poor (16/107, 15%), albeit with only 1% (1/107) reporting a negative attitude toward serving as a control without further notification [21]. Though broad consent is known to have such limitations, it may be a more ethical choice as it reduces clinical and decision-making burden at a vulnerable time in patients’ lives. Nonetheless, dynamic informed consent is a strategy proposed to improve recollection rates whereby patients are actively reminded of their involvement in research by regular study updates and being given the option to either continue or to withdraw [21]. Participants in the NEST feasibility study were sent study questionnaires at regular intervals and were reminded at each trial contact that they had the option to withdraw from the study.

### Strengths and limitations

A strength of this study was the high rate of participation in qualitative interviews by study participants (74%), as well as clinicians and researchers providing a broad range of views. Interviews were carried out by a research team member who was external to the clinical treating team to help participants to speak more freely of their experiences. However, there is a potential for selection bias as those who declined to be interviewed may have done so due to negative experiences of the trial. Due to resource constraints, patients who declined participation in the ceRCT were not interviewed, so barriers to participation in the ceRCT and other elements of the cohort study experience are likely underrepresented thus limiting the generalisability of our results. Furthermore, as only one of two individuals who refused randomisation consented to be interviewed, we do not have data to fully capture these experiences.

The study period spanned the outbreak of the COVID-19 pandemic at which time non-covid related research and treatment of early-stage kidney cancer was deprioritised. This resulted in delays between enrolment and treatment which may have significantly affected patients’ memory recall at the time of interview regarding their experiences of the recruitment, randomisation and consent process. The option for remote interviews via videoconference during the COVID-19 pandemic facilitated interviews at a time when patients were avoiding contact with hospital services. During this time the general population became more familiar with such technology, and this may have facilitated participation amongst those who would have declined to travel to be interviewed and not had the technical know-how to attend remotely in the pre-COVID setting. Furthermore, the study was conducted at a single tertiary-referral hospital in England which may limit the generalisability of the findings to other settings. Whilst saturation was reached for patient interviews, further interviews with clinicians may have been fruitful but were limited by the size of the research group. Further research may benefit from replicating the study interviewing patients prior to randomisation to eliminate any potential bias resulting from a positive treatment outcome.

We suggest the following recommendations for future trials: (1) developing interactive patient information to enhance patient understanding and help clinicians; (2) avoiding the use of clinical or research jargon (e.g. trial) that may cause anxiety or confusion in patients; (3) adopting teach-back methods to assess patients’ understanding prior to consent; (4) employing dynamic informed consent to reinforce patients’ understanding; (5) consider conducting some interviews prior to randomisation to assess perceptions regarding preferences and understanding to reduce potential choice-supportive bias.

## Conclusions

We found that NEST’s ceRCT design was acceptable to patients with high participation rates and positive experiences of participation reported in qualitative interviews. However, our findings must be interpreted with caution due to the possibility of selection, choice-supportive and memory recall biases. Participants valued the pragmatic trial design but reported a preference for more succinct study materials. Clinicians were supportive of the novel, pragmatic trial design, but reported that approaching eligible patients was a challenge in the limited consultation time allowed for a newly diagnosed SRM.

## Data Availability

The datasets used and/or analysed during the current study are available from the corresponding author on reasonable request.
